# Novel Cardiolipins from Uncultured Methane-Metabolizing Archaea

**DOI:** 10.1155/2012/832097

**Published:** 2012-05-14

**Authors:** Marcos Y. Yoshinaga, Lars Wörmer, Marcus Elvert, Kai-Uwe Hinrichs

**Affiliations:** Organic Geochemistry Group, MARUM—Center for Marine Environmental Sciences & Department of Geosciences, University of Bremen, 28359 Bremen, Germany

## Abstract

Novel cardiolipins from Archaea were detected by screening the intact polar lipid (IPL) composition of microbial communities associated with methane seepage in deep-sea sediments from the Pakistan margin by high-performance liquid chromatography electrospray ionization mass spectrometry. A series of tentatively identified cardiolipin analogues (dimeric phospholipids or bisphosphatidylglycerol, BPG) represented 0.5% to 5% of total archaeal IPLs. These molecules are similar to the recently described cardiolipin analogues with four phytanyl chains from extreme halophilic archaea. It is worth noting that cardiolipin analogues from the seep archaeal communities are composed of four isoprenoidal chains, which may contain differences in chain length (20 and 25 carbon atoms) and degrees of unsaturation and the presence of a hydroxyl group. Two novel diether lipids, structurally related to the BPGs, are described and interpreted as degradation products of archaeal cardiolipin analogues. Since archaeal communities in seep sediments are dominated by anaerobic methanotrophs, our observations have implications for characterizing structural components of archaeal membranes, in which BPGs are presumed to contribute to modulation of cell permeability properties. Whether BPGs facilitate interspecies interaction in syntrophic methanotrophic consortia remains to be tested.

## 1. Introduction

One of the most prominent aspects of archaeal biochemistry is the structure of their cellular membrane lipids [1]. Archaeal intact polar lipids (IPLs) are composed of a core lipid (isoprenoidal glycerol diethers and tetraethers) and polar headgroups (phosphoester or sugar-linked headgroups, i.e., phospholipids and glycolipids, resp.). Archaeal membrane lipids can be unequivocally differentiated from other domains of life based on the glycerol backbone stereochemistry [2, 3]. In Archaea, the isoprenoid chains are bound at *sn*-2 and *sn*-3 positions of the glycerol backbone exclusively through ether linkages and linked to a phosphate-based and/or a sugar headgroup attached to the *sn*-glycerol-1 (S configuration). Bacteria and eukaryotes contain headgroups attached to the *sn*-glycerol-3 isomer (R configuration) and core lipids (typically *n*- or methyl-branched fatty acids) bound at *sn*-1,2-diacylglycerol.

**Figure 1:**
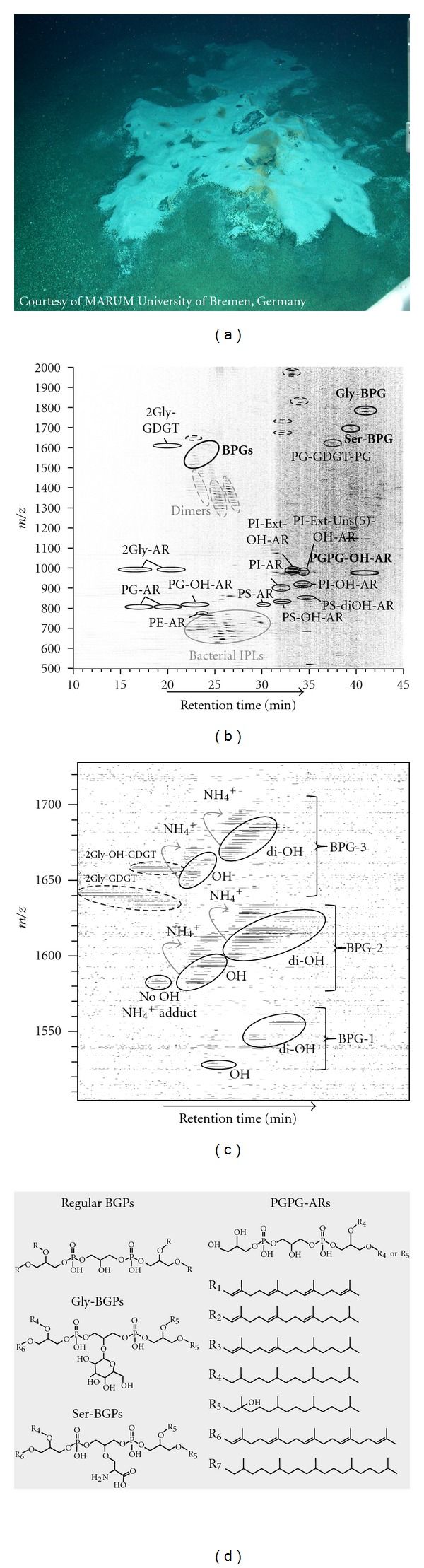
(a) Photography of the seafloor at site GeoB 12315 (~1000 m water depth) during expedition M74/3 taken by the remotely operated vehicle *Quest* (MARUM, University of Bremen) in the continental margin off Pakistan; (b) density map plot showing archaeal IPLs (in black), bacterial IPLs (in gray), and novel archaeal lipids (bold) analyzed in positive mode by HPLC-ESI-IT-MS; (c) zoom in the regular BPGs area of a density map generated in positive mode HPLC-ESI-ToF-MS (BPG-1 with four C_20_, BPG-2 with three C_20_ and one C_25_, and BPG-3 with two C_20_ and two C_25_); (d) structure of archaeal BPGs, other archaeal cardiolipin analogues, and PGPG-ARs. R_1_ to R_7_ are tentatively identified isoprenoidal moieties. *Compound Abbreviations.* GDGT: glycerol dibiphytanyl glycerol tetraether (C_40_–C_40_ isoprenoidal chains); AR: archaeol (C_20_–C_20_ isoprenoidal chains); Ext-AR: extended archaeol (C_20_–C_25_ isoprenoidal chains); OH-AR: monohydroxylated-archaeol (OHC_20_–C_20_); diOH: dihydroxylated-archaeol (OHC_20_–OHC_20_); Uns-AR: unsaturated-archaeol; Gly: glycosyl (hexose); PE: phosphatidylethanolamine; PG: phosphatidylglycerol; PI: phosphatidylinositol; PS: phosphatidylserine; Ser: serine.

A peculiar phospholipid type found exclusively in ATP producing bacterial plasma membranes [4] and the inner membrane of mitochondria [5] is cardiolipin (or bisphosphatidylglycerol, BPG). A unique aspect of BPGs is their dimeric structure constituted by phosphatidic acid linked to phosphatidylglycerol by a phosphoester bond displaying four chains in the hydrophobic tail (Figure 1(d)). Such a structural configuration has implications for the organization of biological membranes, for example, the ability to bind to a large variety of unrelated proteins and the ability to trap protons in energy-converting membranes [6–8]. BPGs and sulfoglycosylated dimeric phospholipids attached to four phytanyl chains have previously been found in extreme halophilic Archaea from natural habitats and cultures [9–11]. In these Archaea, complex dimeric phospholipids are involved in osmoadaptation [12, 13] and cytochrome *c* oxidase activity [14].

Here, we report the structural diversity of novel BPGs and the presence of novel diether lipids in methane-metabolizing Archaea inhabiting surface sediments of methane-charged deep-ocean seeps. These sediments are usually dominated by the uncultured anaerobic methanotrophic (ANMEs) archaea, which are closely associated with sulfate reducing bacteria, jointly performing the anaerobic oxidation of methane (AOM) [15, 16]. This process is not only observed at seeps; AOM is ubiquitous in marine sediments and prevents large amounts of the greenhouse gas methane from escaping into the atmosphere [17]. Analyses of archaeal IPLs coupling high-performance liquid chromatography (HPLC) and ion-trap mass spectrometry (ITMS) from seeps sediments revealed a multitude of diether lipids, including both C_20_–C_20_ archaeol (AR) and C_20_–C_25_ extended AR (Ext-AR) with several combinations of headgroups and presence of hydroxyl group and unsaturation at the isoprenoidal chains [18–20]. Although the diversity and chemotaxonomic relevance of archaeal IPLs from worldwide ANME seep communities have already been examined [19], to date no BPG has been detected in such systems and BPGs have only been restrictedly reported for extreme halophilic archaeal species.

## 2. Material and Methods

In November 2007, during expedition M74/3 onboard the research vessel* Meteor*, the remotely operated vehicle *Quest* (MARUM, University of Bremen) was launched in the continental margin off Pakistan [21]. Sediment cores (ca. 10 cm i.d. and 20 cm length) were recovered from site GeoB 12315-9 (Dive 181) at 1025 m water depth, well within the lower part of the oxygen minimum zone [21]. Surface sediments influenced by gas ebullition were associated with dense microbial mats from sulfide-oxidizing bacteria (Figure 1(a)). Detailed sediment geochemistry and gas emission potential can be found elsewhere [22, 23]. The samples were processed shipboard at 4°C with sediment sections (1-2 cm thick) and immediately placed in liquid nitrogen and later maintained at −80°C at MARUM (University of Bremen, Germany).

The total lipid extract (TLE) was obtained by extraction of 10–20 g wet sediment (0 to 15 cm core depth, 8 samples in total) after addition of 5 *μ*g of internal standard (1-*O*-hexadecyl-2-acetoyl-*sn*-glycero-3-phosphocholine, PAF), using a modified Bligh and Dyer protocol [24]. The TLE was dissolved in a mixture of methanol and dichloromethane (5 : 1 v/v). Initial IPL analysis was performed following conditions previously described [24]. Briefly, chromatographic separation and IPLs analysis were conducted in a ThermoFinnigan Surveyor high-performance liquid chromatography (HPLC) system connected to a ThermoFinnigan LCQ Deca XP Plus ion trap (IT) multiple stage mass spectrometry (MS^n^) equipped with electrospray interface (ESI). A 10 *μ*L aliquot of the TLE (equivalent to 1% of TLE) was injected onto a LiChrospher Diol column (150 × 2.1 mm, 5 *μ*m, Alltech, Germany) equipped with a guard column of the same material. Samples were further analyzed by high-resolution mass spectrometry for precise identification of novel lipids, which allowed mass accuracy in the ppm range. For this purpose, ESI-MS was performed on a Bruker maXis Ultra-High Resolution ToF (ToF) MS. This instrument was coupled to a Dionex Ultimate 3000 UHPLC equipped with a Waters Acquity UPLC Amide column (150 × 2.1 mm, 3.5) following a method recently developed in our lab. IPLs were measured in both positive and negative ionization modes with automated data-dependent fragmentation of base peak ions up to MS^3^ (IT) or MS^2^ (ToF). This method is especially suitable for rapid screening of natural, complex mixtures of membrane lipids with molecular weights in the range from 500 to 2000 Da [24]. Additionally, selected lipids were targeted for MS^2^ fragmentation in multiple reaction monitoring (MRM) mode. In these cases, increasing collision energies (15–55 eV) were applied to the selected parent ion in order to better describe sequential fragmentation.

IPL quantification is semiquantitative (see [24]) and identification is based on mass spectral fragmentation. The quantification and identification of BPGs in samples ran as TLE were hampered by coelution of bacterial phospholipid dimers. To avoid these effects, we purified the analytes of interest by preparative HPLC, using a LiChrospher column (250 × 10 mm, 5 *μ*m, Alltech, Germany) with a fraction collector, as described by [25, 26]. The purified fractions (F1 to F13) were then rerun by HPLC-MS in positive and negative modes to obtain mass spectra on the basis of which BPGs could tentatively be identified in fractions F4 and F12. Given that archaeal IPLs are better characterized by positive ionization mode using our methods [20], only the results from this mode are shown.

## 3. Results and Discussion

Cardiolipin analogues at the station GeoB 12315 were only detected in the upper 10 cm of the sediment column. DNA fluorescent *in situ* hybridization performed with fixed cells at the interval 1-2 cm indicated a dominance of ANME-2 over other methanotrophic archaeal taxa (M. Yoshinaga, K. Knittel, and K.-U. Hinrichs, unpublished data). The structure of BPGs (cf. [9]) and other cardiolipin analogues are described in Figure 1(d). The series of novel dimeric archaeal phospholipids identified tentatively by HPLC-ESI-MS possess masses ranging from 1526.21 to 1684.38 Da for BPGs and up to 1776.36 Da for the other cardiolipin analogues (Table 1). While the BPGs were minor components representing less than 0.5% of total archaeal IPLs, the glycosylated dimeric phospholipids represented 3–5% of total archaeal IPLs in the first 5 cm of the sediment column.

### 3.1. BPGs

Tentative identification of BPGs was achieved by interpretation of exact masses of both parent ions and fragments. In order to be considered for identification, the difference between calculated and measured mass (Δ*m* = (*m*/*z*_measured_ − *m*/*z*_calculated_)/*m*/*z*_calculated_) had to be below 3 ppm for parent ions and 5 ppm for fragments. Similarly to bacterial BPGs [24], molecular ions of BPGs occur both as protonated and as ammonium adducts in HPLC-MS ([M + H^+^]^+^ and [M + NH_4_^+^]^+^, Figure 1(c)).

**Figure 2:**
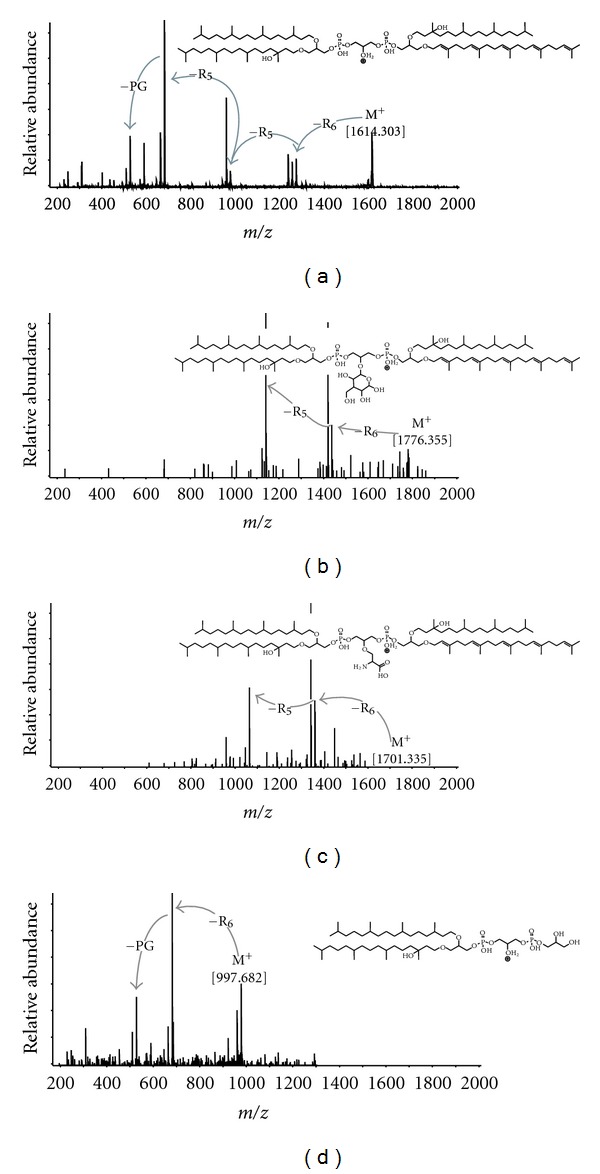
Positive mode MS^2^ spectra of novel archaeal IPLs analyzed by HPLC-ESI-MS. (a) Representative BPG (OHC_20:0_/OHC_20:0_/C_20:0_/C_25:5_-BPG); (b) glycosylated BPG; (c) serine BPG; (d) PGPG-OH-AR. Losses are indicated by *R_n_* (Figure 1(d)). Detailed information on major ions in MS^2^ spectra is available in Table 1.

As illustrated in Figure 1(c), BPGs can be further divided into three major groups, containing the following combinations of isoprenoidal chains: (i) BPG-1 with four C_20_; (ii) BPG-2 with three C_20_ and one C_25_; (iii) BPG-3 with two C_20_ and two C_25_ (see Table 1 for detailed information).  Figure 2(a) shows the fragmentation pattern for a representative BPG, precisely the OHC_20:0_/OHC_20:0_/C_20:0_/C_25:5_-BPG ([M + H^+^]^+^, *m/z* 1614.30). MS^2^ spectra showed major fragment ions at *m/z* 1273.9, 977.6, and 681.3, matching consecutively, the loss from the molecular ion of one penta-unsaturated sesterpenyl (340.3 Da loss) and two OH phytanyl chains (296 Da loss). Other minor fragments observed in MS^2^ (Figure 2(a) and Table 1) are attributed to the fragment ion of the PG headgroup attached to a glycerol backbone containing a single phytanyl chain (*m/z* 527.3) and the subsequent losses of the glycerol headgroup and the phytanyl chain (*m/z* 435.3 and 247.0), which are commonly observed in PG-based AR fragmentation during ESI-MS [20]. The exact mass analysis of fragmentation patterns of this compound is thus consistent with the proposed structure and allowed to constrain the distribution of double bonds and OH moieties on the isoprenoidal chains. Similar MS^2^ spectra were constructed for other BPGs and some general patterns of hydroxyl group and double bond distribution could be established (Table 1). Only phytanyl moieties were observed with one hydroxyl group per chain, while unsaturation occurred on both C_20_ and C_25_ nonhydroxylated isoprenoidal chains. These structural features of isoprenoidal chains resemble those from phosphobased ARs and Ext-ARs described earlier [20] in seep sediments.

Concerning fragmentation patterns, BPGs previously identified in halophilic archaea were analyzed by ESI-MS in negative mode [9–11], among other techniques. For example, tetra C_20:0_-BPG fragmentation is characterized by the cleavage of the diphytanyl-glycerol-phosphate from the molecular ion at *m/z* 1521.3, yielding major fragment ions at *m/z* 805.6, 731.6, and 433.3, corresponding to quasi-molecular ions of PG-AR, phosphatidic acid (PA)-AR and the loss of one phytanyl chain plus water from PA-AR, respectively (e.g., [9]). Our experiments showed prominent fragmentation resulting primarily from the loss of isoprenoidal chains, with minor fragments attributed to the cleavage of the phosphatidylglycerol headgroup (Figure 2(a) and Table 1). Previously described archaeal BPGs were solely composed of four phytanyl chains [9–11]; this structural diversity has now been extended by our findings of novel derivatives (Table 1). The tentative structural assignments of archaeal BPGs by positive ion mode HPLC-ESI-MS are generally consistent with fragmentation patterns of archaeal IPLs. For example, fragmentation of phospho-ARs, such as PG-AR, is dominated by the loss of the head group, so that the MS^2^ spectra display major fragment ions at *m/z* 733.6 (PA-AR). By contrast, phosphobased OH-ARs present a 296.3 Da loss corresponding to the cleavage of the hydroxylated phytanyl chain, which is favored over loss of head group or nonhydroxylated phytanyl chain [20]. Similarly, BPGs in our samples undergo primary loss of the alkyl substituent in a systematic fashion: unsaturated over hydroxylated over saturated isoprenoidal chains (Figure 2 and Table 1). These patterns are reflected in major fragment ions in MS^2^ experiments. Furthermore, when isoprenoidal chains are lost, the initially formed fragment ion is accompanied by fragments resulting from additional losses of water molecules (Figure 2(a)).

### 3.2. Other Cardiolipin Analogues

The same combination of OHC_20:0_/OHC_20:0_/C_20:0_/C_25:5_ isoprenoidal chains was observed for both complex glycosylated and serine cardiolipin analogues (Gly-BPG and Ser-BPG, Figure 1(b)). The Gly-BPG is analogous to the glycosylated cardiolipin from the group B *Streptococcus* strains [27] and structurally distinct from the glycocardiolipin described in earlier studies of archaeal cardiolipin analogues [9–11]. The inclusion of a serine in the central glycerol of BPGs is similar to the D-alanyl and L-lysyl cardiolipins described, respectively, by [28, 29]. Gly-BPG and Ser-BPG showed molecular ions in MS^1^ mode corresponding to the protonated and the ammonium adduct (Figure 1(b)). Fragmentation pattern in MS^2^ is also similar, with major fragment ions observed from loss of a penta-unsaturated sesterpenyl chain (340.3 Da) and a subsequent OH phytanyl (296.3 Da) loss (Figures 2(b) and 2(c)). Minor fragment ions include *m/z *977.6 and 959.6, which can also be observed in typical BPGs (Table 1) and are consistent with the successive losses of penta-unsaturated sesterpenyl and OH phytanyl chains together with the hexose or serine.

### 3.3. Novel Archaeal Diether Phospholipids

In addition to cardiolipin analogues, two novel archaeal diether lipids were detected and characterized by HPLC-ESI-MS. These compounds are structurally related to the BPGs, with the difference that they contain only two isoprenoidal chains (Figure 1(d)). The fragmentation of the tentatively identified bisphosphatidylglycerol archaeol or PGPG-AR in MS^2^ experiments ([M + H^+^]^+^, *m/z *961.68) is marked by the loss of the glycerol headgroup (74.0 Da) plus water and the loss of PG (154.0 Da), resulting in major fragment ions at *m/z* 869.6 and 807.6, respectively (Table 1). Minor fragment ions at *m/z* 733.6 and 435.3 are identical to those observed for PG-AR in MS^2^ positive ion mode [20]. The tentatively identified PGPG-OH-AR ([M + H^+^]^+^, *m/z *977.68) undergoes a prominent loss of 296.3 Da and a subsequent 74.0 Da loss, yielding major fragment ions at *m/z* 681.3 and 527.3 (Figure 2(d)), which can be also observed as minor fragments during MS^2^ experiments of BPGs (Table 1). These novel compounds represent less than 1% in the 1–11 cm and increase to 3% of total archaeal IPLs at the 11–15 cm horizons (data not shown).

After careful reinspection of samples dominated by ANME-2 archaea [17, 18], PGPG-OH-AR was exclusively detected in Black Sea microbial mats, Arabian Sea, and Hydrate Ridge seep sediments. Given the structural resemblance of PGPG-ARs and typical BPGs (Figure 1(d)) and their occurrence pattern restricted to seep sediments with high ANME-2 abundance, we hypothesize that these diether lipids are likely degradation products of the BPGs. However, one cannot rule out the participation of these diethers as intermediates in archaeal BPGs biosynthesis, which is still unknown.

### 3.4. Possible Significance of Novel Archaeal Cardiolipin Analogues in Methane-Metabolizing Archaea

Archaeal diether and tetraether IPLs generally contain saturated isoprenoidal chains (e.g., [30] and references therein). The only archaeal BPGs described so far are invariably composed of saturated phytanyl chains [9, 11]. In our samples, we observed that cardiolipin analogues are structurally more complex and attached to multiple combinations of saturated, hydroxylated, and polyunsaturated C_20_ and C_25_ isoprenoidal chains (Table 1). Unsaturated isoprenoids are found in archaeal isolates over a wide temperature range, for example, thermophilic [31, 32] and psychrophilic [33, 34], so that unsaturation of isoprenoidal chains is probably not primarily a membrane adaptation to temperature [35]. Given that the physical stability of isoprenoidal chains is the major regulating factor for low proton permeability in archaeal liposomes [36], unsaturation of isoprenoidal chains results in increased solute permeability through the cell membrane. Indeed, unsaturation appears to be widespread among halophilic archaea [33, 34, 37], the only cultivated BPG producers [38]. In addition, an increase in cardiolipin analogue content was observed in halophilic archaea when exposed to low salt conditions [12, 13]. In cold seep sediments, both core lipids (i.e., AR and OH-AR) and IPLs are relatively well characterized (e.g., [19, 39]), but thus far neither BPGs nor isoprenoidal chain unsaturation has been reported as an important feature, except for minor amounts of the recently described phosphobased unsaturated ARs [20].

The asymmetric arrangement in archaeal BPGs, that is, C_20_ and C_25_ isoprenoidal chains, including the presence of unsaturations and/or hydroxyl-group(s) (Table 1), differs from the prevalent symmetric patterns of mitochondrial cardiolipins [40]. In addition, because cardiolipins are typically minor lipids in bacterial and mitochondrial membranes, besides the fact that no clear pattern of cardiolipin unsaturation is found among different organisms, they are not believed to affect overall fluidity of the cellular membrane [7, 40]. The high diversity of typical BPGs and the relatively high abundance of the complex glycosylated and serine cardiolipin analogues in concert with the extensive presence of unsaturation in the isoprenoidal chains have implications for the bioenergetics of membrane lipids. First, an increase in proton permeability could facilitate archaeal catabolism in microbial communities mediating the anaerobic oxidation of methane, a process known to yield minimal metabolic energy [41]. Second, among the two most widespread ANME groups, ANME-2 representatives are putatively found physically associated with sulfate-reducing bacteria in cluster-like arrangements [16], whereas ANME-1 often occur as single cells (e.g., [42–44]). Under the assumption that BPGs and PGPG-ARs are affiliated with ANME-2 archaea, it is conceivable that interaction between cardiolipin analogues and membrane proteins facilitates the transport of protons, electrons, and/or metabolites (similarly to mitochondria or ATPase/synthase bound cardiolipins, e.g., [6, 8, 14]) in the cell-to-cell syntrophic surroundings.

In this study, we have tentatively identified several novel archaeal cardiolipin analogues on the basis of their fragmentation patterns during positive ion mode HPLC-ESI-MS. As methane-metabolizing archaea are yet to be isolated in culture, investigations on the function of cardiolipin analogs in Archaea should proceed with detailed lipid examination of already cultured species.

## Figures and Tables

**Table 1:** Characterization of novel PG-based ARs and BPGs with representative fragmentation patterns observed (for abbreviations see text). Sequential losses of alkyl chains are shown together with fragmentation of the headgroup when remaining attached to the glycerol backbone containing a C_20:0_ chain. Values in brackets indicate loss from the most direct precursor, not from the parent ion; (*) denotes presence of unsaturation or OH-group in isoprenoidal chains, which were not exactly determined by MS^2^ spectra; **loss of OH-phytanyl chain and hexose from the headgroup; (***) loss of serine plus water from the headgroup with two chains still attached (see Figure 2). Fragments due to losses of alkyl chains are coded: underscored (fragment resulting from loss of an unsaturated chain), bold (fragment resulting from loss of C_25_ chain) and *italics* (fragment resulting from loss of a hydroxylated phytanyl chain). Additional losses of H_2_O occur in hydroxylated alkyl moieties, but are not specified here. All parent ions identified show Δ*m* < 3 ppm; all fragments identified have Δ*m* < 5 ppm. Δ*m* = (*m*/*z*_measured_ − *m*/*z*_calculated_)/*m*/*z*_calculated_.

	*m*/*z *	Chain characterization				Fragmentation due to losses of chains (loss of)				Headgroup attached to glycerol containing a C_20:0_ chain (loss of)				GPG fragment
						1st chain	2nd chain	3rd chain	4th chain	C_3_H_8_O_3_	PO_2_	C_3_H_8_O_3_	PO_2_	
PGPG-AR	961.687	C_20:0_	C_20:0_	—	—	681.374 (280.3)				589.326 (92.0)				

PGPG-OH-AR	977.682	OH–C_20:0_	C_20:0_	—	—	*681.374 (296.3)*				589.326 (92.0)	527.370 (62.0)	435.324 (92.0)	355.357 (62.0)	

OH BPG-1	1538.308	OH–C_20:0_	C_20:0_	C_20:0_	C_20:0_									
	1526.214	C_20:3_	C_20:3_	OH–C_20:0_	C_20:0_	1251.948 (274.3)	977.682 (274.3)							

diOH BPG-1	1554.303	OH–C_20:0_	OH–C_20:0_	C_20:0_	C_20:0_	*1257.995 (296.3)*	*961.687 (296.3)*							
	1544.224	*OH–C_20:0_	*OH–C_20:0_	*C_20:3_	*C_20:2_									

BPG-2	1581.199 (NH_4_^+^)	*C_25:5_	*C_20:3_	*C_20:3_	*C_20:3_				**401.061 ** **(1180.1)**					

OH BPG-2	1598.308	C_25:5_	OH–C_20:0_	C_20:0_	C_20:0_	**1257.995 ** **(340.3)**	*961.687 (296.3)*							
	1594.277	C_25:5_	C_20:2_	OH–C_20:0_	C_20:0_	**1253.963 ** **(340.3)**	977.682 (276.3)							
	1592.261	C_25:5_	C_20:3_	OH–C_20:0_	C_20:0_	**1251.948 ** **(340.3)**	977.682 (274.3)	*681.374 (296.3)*						
	1582.183	C_25:5_	C_20:4_	C_20:4_	OH–C_20:0_			**697.369 ** **(884.8)**	*401.061 (296.3)*					

diOH BPG-2	1624.381	OH–C_20:0_	OH–C_20:0_	C_25:0_	C_20:0_	*1328.073 (296.3)*	*1031.765 (296.3)*							
	1614.303	C_25:5_	OH–C_20:0_	OH–C_20:0_	C_20:0_	**1273.990 ** **(340.3)**	*977.682 (296.3)*	*681.374 (296.3)*	401.061 (280.3)	589.326 (92.0)	527.370 (62.0)	435.324 (92.0)		247.058
	1608.256	C_25:5_	OH–C_20:3_	OH–C_20:0_	C_20:0_	**1267.947 ** **(340.3)**	*977.682 * *(290.3)*	*681.374 (296.3)*		589.326 (92.0)	527.370 (62.0)	435.324 (92.0)		247.058

OH BPG-3	1668.39	*C_25:5_	*C_25:5_	*OH–C_20:0_	*C_20:0_									
OH BPG-3	1652.261	C_25:5_	C_25:5_	C_20:3_	OH–C_20:0_	**1311.948 ** **(340.3)**	1037.682 (274.3)	**697.369 ** **(340.3)**	*401.061 (296.3)*					

diOH BPG-3	1684.381	C_25:5_	OH–C_20:0_	OH–C_20:0_	C_25:0_	**1344.068 ** **(340.3)**	*1047.760 (296.3)*	*751.452 (296.3)*						
	1676.318	C_25:5_	C_25:4_	OH–C_20:0_	OH–C_20:0_	**1336.005 ** **(340.3)**	**933.677 ** **(340.3)**	*697.369 (296.3)*	*401.061 (296.3)*					
	1674.303	C_25:5_	C_25:5_	OH–C_20:0_	OH–C_20:0_	**1333.990 ** **(340.3)**	**933.677 ** **(340.3)**	*697.369 (296.3)*	*401.061 (296.3)*					
	1670.271	C_25:5_	C_25:4_	OH–C_20:3_	OH–C_20:0_		**987.630 ** **(682.6)**	*697.369 * *(290.3)*	*401.061 (296.3)*					

Gly-BPG	1776.355	C_25:5_	OH–C_20:0_	OH–C_20:0_	C_20:0_	**1436.042 ** **(340.3)**	*1139.734 (296.3)*	681.373** (296.3 + 162.1)						

Ser-BPG	1701.335	C_25:5_	OH–C_20:0_	OH–C_20:0_	C_20:0_	**1361.022 ** **(340.3)**	*1064.714 (296.3)*			959.671 (105.0)***				
